# New Burnout Evaluation Model Based on the Brief Burnout Questionnaire: Psychometric Properties for Nursing

**DOI:** 10.3390/ijerph15122718

**Published:** 2018-12-02

**Authors:** María del Carmen Pérez-Fuentes, María del Mar Molero Jurado, África Martos Martínez, José Jesús Gázquez Linares

**Affiliations:** 1 Department of Psychology, Faculty of Psychology, University of Almería, 04120 Almería, Spain; mmj130@ual.es (M.d.M.M.J.); amm521@ual.es (Á.M.M.); jlinares@ual.es (J.J.G.L.); 2 Department of Psychology, Universidad Autónoma de Chile, Región Metropolitana, Providencia 7500000, Chile

**Keywords:** burnout, psychometric properties, nursing

## Abstract

Health care personnel are considered one of the worker sectors most exposed to heavier workloads and work stress. One of the consequences associated with the exposure to chronic stress is the development of burnout syndrome. Given that evaluating this syndrome requires addressing the context in which they are to be used, the purpose of this work was to analyze the psychometric properties and structure of the Burnout Brief Questionnaire (CBB), and to propose a more suitable version for its application to health professionals, and more specifically nurses. The final study sample was made up of 1236 working nursing professionals. An exploratory factorial analysis was carried out and a new model was proposed through a confirmatory factorial analysis. Thus, validation of the CBB questionnaire for nursing health care personnel showed an adequate discrimination of the items and a high internal consistency of the scale. With respect to the factorial analysis, four factors were extracted from the revised model. Specifically, these new factors, called job dissatisfaction, social climate, personal impact, and motivational abandonment, showed an adequate index of adjustment. Thus, the Brief Burnout Questionnaire Revised for nursing staff has favorable psychometric properties, and this model can be applied to all health care professionals.

## 1. Introduction

The number of health care workers in Spain increases year after year, as the number of official association members testifies, in fields such as medicine, which in 2015 increased by 1.9%. The number of nurses rose by 3.4% [1] to nearly 300,000 registered nurses according to the latest data from the National Statistics Institute [2]. Furthermore, the role of nursing personnel is more and more important, and the emotional skills and stressful work climate of nurses must be taken into account, but not only for them, as there are now studies analyzing these factors even in students of physiotherapy, for example [3]. Therefore, nurses are gradually facing situations and settings with more pressure and heavier workloads [4], which produce scenarios filled with strain and job stress [5].

According to the Encyclopedia of Mental Health, burnout syndrome is a type of response to chronic emotional and interpersonal stress factors at work, which is recognized as a serious occupational hazard [6]. The presence of stressors at work maintained over long periods of time can cause burnout in workers, especially those who maintain a constant direct care relationship with service users, as is the case of health care personnel [7], although this syndrome may also be discussed in other areas [8,9,10].

The presence of burnout syndrome in workers leads to physical, occupational, and psychological consequences, in particular, cardiovascular problems, pain, depressive symptoms, sleep problems, alcohol abuse, absenteeism, and job dissatisfaction [11]. Its appearance has also been associated with a multitude of individual and psychosocial variables [12,13].

One of the behaviors associated with this syndrome is demotivation [5,14]. Specifically, the deterioration of professional motivation, which affects almost half of nursing personnel [15], is a process derived from the perception of absence of reward and culminates in the individual’s depersonalization [16]. Motivation, which refers to choices of ends and means, depends in large part on an individual’s beliefs and values at the time a situation is evaluated. Motivation generates feelings that lead to action on the job, while demotivation creates limits and promotes expressions of displeasure and distress [17]. According to a study by Achour et al. [18], lack of recognition and motivation are two challenges that health care personnel must face as heavier workloads are assigned and measurements of performance become stricter. This directly affects their performance and job satisfaction [19]. So nursing professionals with the most intrinsic motivation (that is, motivated by their own enjoyment of performing the task for humanitarian reasons) and extrinsic motivation (associated with economic characteristics and scheduling flexibility) show higher levels of job satisfaction and less burnout [20].

Job satisfaction specifically refers to the enjoyment individuals find in their job [21]. Lack of satisfaction in health care jobs has been associated with the presence of burnout in workers, and also with the intention of quitting the profession and diminishing quality of the care given [22,23]. According to Farnaz et al. [24], job satisfaction in nursing is associated mainly with environmental factors, in detriment to sociodemographic and individual factors, so improving satisfaction in job positions involves enriching the characteristics of the organizations nurses work in.

The workplaces with the highest quality, with regard to both setting and structure, are associated with greater well-being and lower levels of burnout among health care personnel [25,26]. Therefore, it is of vital importance that healthy work environments, where the psychological health of nursing staff is given attention, be promoted [27]. In turn, study of the prevention, treatment, and measurement of severe widespread problems in this population, such as burnout syndrome, must continue to progress [28].

The most widely used instrument for the evaluation of burnout is the Maslach Burnout Inventory (MBI) [29]. This instrument was designed for evaluating professionals, such as nurses, who perform their job while interacting with users of their service [30], and has been extensively described and validated internationally [31]. Its manual describes burnout as occurring at high levels of emotional exhaustion and depersonalization, in combination with low scores in personal accomplishment. However, other studies [32] make use of alternative proposals to determine the presence of burnout, such as the definition by Poncet et al. [33], who estimated that this syndrome is present among professionals with a cumulative score over –9 on the MBI. 

Although there are studies confirming the Maslach Burnout Inventory questionnaire’s three dimensions [34,35,36], other studies have found factor structures based on two [37,38] and five dimensions [39,40]. Densten [39], after confirmatory analysis of the instrument, found that the structure based on five scales was more strongly supported than the model of three, or even four. Thus, while the depersonalization factor was maintained in this new division of factors, emotional exhaustion was divided into “somatic strain” and “psychological strain,” while personal accomplishment was broken down into “self-accomplishment” and “working with others.” 

Another alternative instrument to the MBI for evaluating burnout is the Brief Burnout Questionnaire (CBB) [41]. The CBB comprises 21 items that evaluate not only the syndrome itself, but also its antecedents and consequences. That is, it understands burnout as a process [42]. The instrument was validated in teaching professionals, showing adequate convergent validity with the MBI on the total burnout scale (but not, however, on all the syndrome factors), so the authors recommended it for evaluating some elements present in the burnout process (specifically antecedents, burnout, and consequences), but not for direct evaluation of its specific components. Few studies have used this questionnaire [43,44,45], as shown in the review by Ahola et al. [46], who indicated that they were unaware of the existence of the questionnaire’s validation. It has also been adapted for use with housewives [47], in which a three-factor structure similar to the one found in the original questionnaire was found. However, this instrument has received some criticism. For example, in the validation done in a sample of teachers in Aragon Province, Spain [48], no significant differences were found on some of the scales between men and women, which might be due to the inappropriateness of the items in showing the behavior associated on each scale. The results have also shown low reliability, and it was concluded that its use has generated little validity, mainly because of its factor division [49]. 

According to Domínguez-Lara [50], the multifactor internal structure of burnout evaluation instruments must be analyzed considering the context where they are going to be used, since even though the construct may have a good theoretical basis, the configuration of its structure may vary when used in real environments. Therefore, the purpose of this study is to show that the CBB is a valid model for different cultures and societies, as this scale has awakened great interest in recent years. In addition to analyzing its psychometric properties and structure, this study proposes the best version or model for its application to health care professionals, and nurses in particular. 

## 2. Materials and Methods

### 2.1. Participants

The sample was made up of 1352 nurses selected at random from several health centers, and therefore actively employed at the time data were collected. Subjects who did not complete the questionnaire or who gave random answers (detected by control questions) were eliminated from the study. The final sample consisted of a total of 1236 participants, of whom 69.3% (*n* = 857) were working under temporary contracts and the other 30.7% (*n* = 379) had permanent contracts.

The mean participant age was 31.50 years (*SD* = 6.18), in a range of 21 to 57 years. Of the whole sample, 84.5% (*n* = 1044) were women and 15.5% (*n* = 192) were men, with a mean age of 31.65 years (*SD* = 6.23) and 30.71 years (*SD* = 6.17), respectively. Their marital status was 55% (*n* = 680) single, 42.1% (*n* = 520) married or in a stable relationship, 2.8% (*n* = 34) divorced or separated, and 0.2% (*n* = 2) widowed. In addition, 68.9% (*n* = 852) of the participants had no children, 14.5% (*n* = 179) had 1 child, 13.2% (*n* = 163) had 2 children, and the remaining 3.3% (*n* = 41) had 3 or more.

Their distribution by area of work was 32% (*n* = 396) staff nurse and 21.9% (*n* = 271) on emergency teams, while 11.4% (*n* = 141) were working in the ICU, 10.7% (*n* = 132) in surgery, 2.3% (*n* = 28) in outpatient care, and 4% (*n* = 50) in the mental health unit. The remaining 17.6% (*n* = 218) were working in other areas.

### 2.2. Instruments

An ad hoc questionnaire was prepared to collect sociodemographic data (age, sex, marital status, and degree) and to compile information on their profession and work experience: years of experience, employment situation (permanent or temporary), work shifts (rotating, 12 h or more, nights only, and morning/afternoon), and number of patients attended to in a workday. 

The CBB [41] was used to evaluate burnout syndrome in the professionals. This instrument consists of 21 items in three blocks corresponding to antecedents of burnout and its elements and consequences. Even though the purpose of the questionnaire is to evaluate the professional burnout process, it includes factors proposed in the Maslach and Jackson model [29] and components that precede and support it. The answer format is a 5-point Likert-type scale. Items 2, 4, 8, 9, and 16 must be inverted and recoded after inversion to find the corresponding overall subscale scores. 

### 2.3. Procedure

Before the data were collected, compliance with participant information standards, confidentiality, and ethics in data processing was guaranteed. Questionnaires were implemented on a Web platform, which enabled participants to fill them out online. A series of control questions were included to detect chance or incongruent answers, and any such cases were discarded from the study sample.

### 2.4. Data Analysis

**Figure 1:**
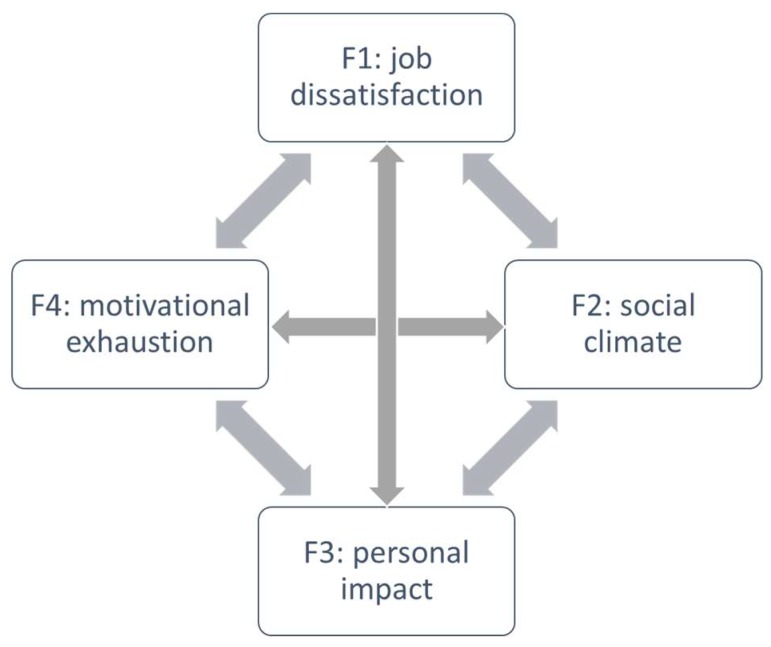
Proposed Burnout model.

The descriptive and confirmatory data analyses were done following the steps by Pérez-Fuentes et al. [51]; in addition, validation was performed in two stages following the steps by Álvarez-García et al. [52]. The first stage was intended to study the structure of the CBB. To approach this objective, the sample was first randomly divided into two independent homogeneous subsamples. The first (*n* = 605) was used as a calibration sample for confirmatory factor analysis (CFA) of the burnout model proposed (Figure 1). Then CFA was done for the proposed model, taking the following fit indices as measures: *χ^2^/gl*, comparative fit index (CFI), Tucker–Lewis index (TLI), root mean square error of approximation (RMSEA), with the confidence interval (CI) at 90%. The index *χ^2^/gl* was used considering values below 5 acceptable [53], Comparative Fit Index (CFI) and Incremental Fit Index (IFI) over or near 0.95, and RMSEA below or very near 0.06 [54]. As a general rule, good fit of the model would be found when ratio 2/GL (degrees of freedom)≤ 3; Goodness-of-fit index (GFI), adjusted goodness of fit index (AGFI), and TLI > 0.90; CFI > 0.95; and RMSEA ≤ 0.05. The advisable respecifications were made to the proposed model, which showed good fit indices, considering theoretical and statistical criteria (modification indices, estimation errors, standardized errors of measurement). The Akaike information criterion [55] was used for model selection based on the second subsample (*n* = 635), which was used as the validation sample to validate the respecified model. Cronbach’s alpha [56] and split halves were used for the reliability analysis of the new scale.

In the second stage, an analysis was done to support the proposed invariant factor structure across type of contract (permanent or temporary) and gender (male/female). First, both subsamples were checked to see the goodness of fit of these structures (model M0a, Permanent-Male, and model M0b, Temporary-Female). The four resulting nested models were evaluated: (a) Model 1. Both subsamples were considered simultaneously with free parameter estimation. (b) Model 2. Metric invariance was demonstrated. (c) Model 3. Scalar invariance was demonstrated. (d) Model 4. Strict invariance. There was no consensus criterion to determine the criteria to be used to evaluate the difference in fit of the nested models [57]. This study used ΔCFI to evaluate its fit. ΔCFI interprets the model as fully invariant if the value found is below 0.01 [58]. The analyses were performed using the SPSS version 23.0 Statistical Package for Windows (IBM, Armonk, NY, USA) and the AMOS 22 program (IBM, Armonk, NY, USA).

## 3. Results

### 3.1. Preliminary Analyses

In the first place, the data show that the CBB items have a normal distribution according to the criterion of Finney and DiStefano [59], who give 2 and 7 as the maximum permissible for skew and kurtosis, respectively. In our study, the maximum was 1.24 and 2.15, respectively. In the exploratory factor analysis, principal component extraction was used with direct oblimin rotation (KMO = 0.85), which allows correlation between factors. Based on the exploratory analysis and various previous studies on validation of the questionnaire itself, other versions, and previous research, a new model is proposed.

### 3.2. Exploratory Factor Analysis of the Original CBB Model

The principal component analysis (chosen since the determinant of *p* = 0.086 showed intercorrelation of the variables, required for this method) revealed the existence of two components with eigenvalues over 1 in the first block, that is, the general antecedents scale. Thus, the scree test indicated the advisability of rotation with two factors with eigenvalues of 3.56 and 1.37, since they were clearly distanced from the third, with a score of 0.86. 

After factor analysis, the items with factor saturations over 0.40 were selected from the direct oblimin rotation matrix of rotated components. As seen in Table 1, factor 1 corresponds to the items that make up the scale’s organization factor. Factor 1 comprises four items, all with loadings over 0.60, which explain 38.18% of the variance. Factor 2 is made up of five items and forms part of the task component, and explains 15.22% of the variance.

In the second block of the burnout syndrome scale, principal component analysis (determinant *p* = 0.124 shows intercorrelation of the variables) revealed the existence of one component with an eigenvalue over 1. As the theoretical structure of the construct was three factors, we used principal axis factoring to force the presence of three factors with varimax rotation. The scree test shows the adequacy of rotation with one factor with a value of 3.38, and the following two are scarcely below 1, with values of 0.98 and 0.96, although they are at a distance from the quartile score of 0.84. 

After the factor analysis, we selected the items with the highest factor saturations from the matrix of rotated components (varimax rotation). Table 2 shows how factor 1 corresponds to the items that make up the scale’s emotional exhaustion factor. This factor comprises three items, all with loadings over 0.60, explaining 31.93% of the variance. The original questionnaire did not include item 3 in this factor, which saturated highest in factor 3. Factor 2 comprises four items, which form the lack of accomplishment component, explaining 4.99% of the variance. Item 18 is included in this factor, but not in the original version, where it was in factor 3. Finally, it should be mentioned that factor 3, which is formed by the depersonalization component, is composed of two items, and that item 3 is in this factor, unlike the original questionnaire.

The third part of the scale corresponds to the consequences of burnout, and analysis of principal components revealed the existence of one component with eigenvalues over 1. It comprises three items (13, 17, and 21), all with loadings over 0.75 (.79, 0.79, and 0.76, respectively), which explain 60.53% of the variance (KMO = 0.66; *χ*^2^_(3)_ = 560.17, *p* < 0.000; Cronbach’s alpha = 0.67).

### 3.3. Exploratory Factor Analysis of Revised CBB Model (CBB-R)

Principal component analysis (chosen because the determinant of *p* = 0.001 shows intercorrelation of the variables, required by this method) revealed the existence of four components with eigenvalues over 1. The scree plot recommends rotating with four factors, with eigenvalues of 3.56 and 1.37, respectively, as they are at a clear distance from the third with a score of 0.86.

After factor analysis, we selected the items with factor saturations over 0.40 from the direct oblimin rotation matrix of rotated components. As seen in Table 3, factor 1 corresponds to the items that make up the scale’s job dissatisfaction factor. This factor comprises five items, all with loadings over 0.55, explaining 31.77% of the variance. Factor 2 has four items that form the social climate component, explaining 8.41% of the variance. Factor 3 has seven items, which make up the personal impact component and explain 5.05% of the variance. Finally, factor 4 (five items) is related to motivational exhaustion.

### 3.4. Confirmatory Factor Analysis of CBB Model and CBB-R Model

Table 4 analyzes the fit of the various models of the questionnaire by the original CBB model, the unidimensional CBB model, the proposed four-factor CBB model, and the revision of that model first proposed (CBB-R). The original model and the unidimensional model show values that are not very adequate. The proposed four-factor CBB model, which corresponds to what was found in the exploratory analysis, is better, but while it showed good fit indices, the advisable respecifications were made considering theoretical and statistical criteria (modification indices, errors of estimation, standardized errors of measurement), which led to elimination of items 2, 16, 3, 13, 17, and 11. The revised model showed much better fit with the calibration sample. The difference between the AIC default model value (248.497) and the AIC saturated model value (240.000) is also very small, showing that this is probably the best of the models according to the Akaike model selection criteria. 

**Figure 2:**
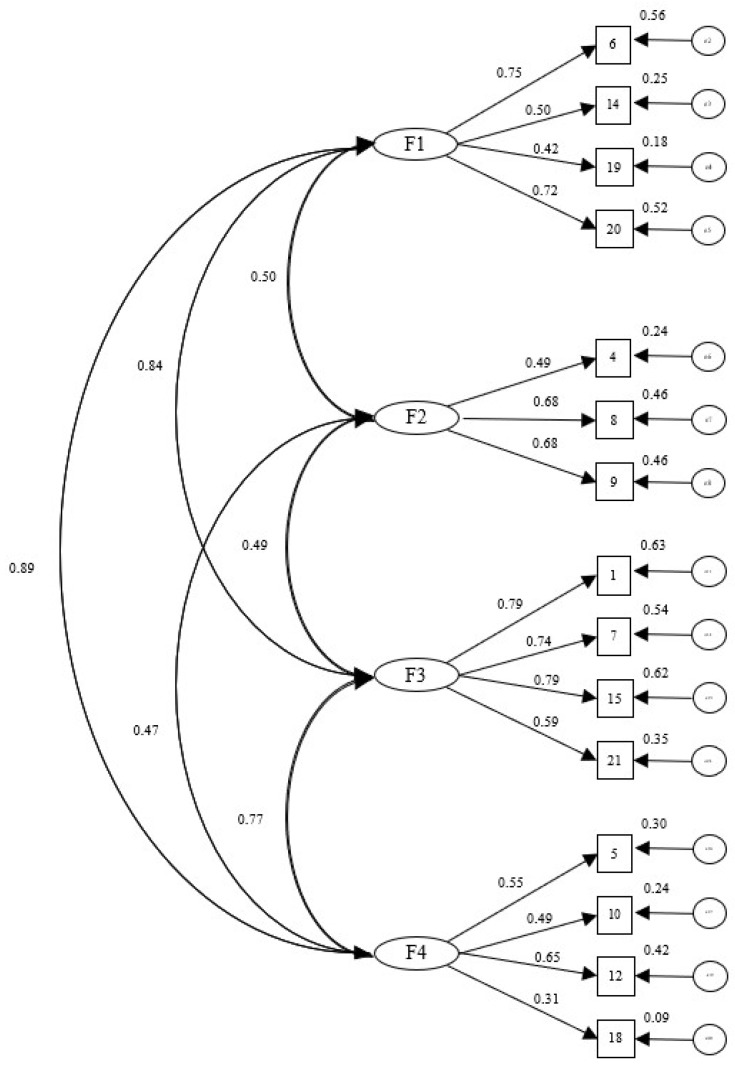
Proposed CBB-R model (validation sample *n* = 635). Note: F1: job dissatisfaction; F2: social climate; F3: personal impact; F4: motivational exhaustion.

Fit indices for the proposed CBB-R model with the validation sample (*n* = 635) are shown in Figure 2. Confirmatory factor analysis for the proposed model was done, taking the following fit indices as measures: *χ*^2^/*gl =* 2.241, CFI = 0.961, TLI = 0.951, RMSEA = 0.044 (0.036–0.053). 

The reliability of the model was analyzed using Cronbach’s alpha, where α = 0.89 for the total sample; for factor 1 (job dissatisfaction), comprising four items, α = 0.697; for factor 2 (social climate), made up of three items, α = 0.666; for factor 3 (personal impact), made up of four items, α = 0.808; and for factor 4 (motivational exhaustion), comprising four items, α = 0.529. Furthermore, the data found by split halves also showed both equal-length (Spearman–Brown coefficient = 0.818) and unequal-length (Spearman–Brown coefficient = 0.819) consistency of the scales.

Table 5 shows the values for all six models. It can be seen how ΔCFI is over 0.01 for models 3 and 4, accepting the configural and metric invariance. Specifically, ΔCFI between model 1 (configural and metric base model) and models 3 and 4 is 0.024, so scalar and strict invariance cannot be accepted. In the analysis of variance by gender, in all cases ΔCFI is under 0.01, so the configural, metric, scalar, and strict invariances are accepted.

## 4. Discussion

The validation of the CBB questionnaire for health care personnel in nursing shows adequate discrimination of items. Cronbach’s alpha for this scale was 0.089, which shows its high internal consistency.

With respect to the factor analysis, four factors were extracted from the revised model, which differed from the original structure of the Brief Burnout Questionnaire [41]. This model was proven to generate better fit of the data than the original. The percentage explained by this model was 51.86%, emphasizing the first factor, where all the items loaded over 0.55 and explained 31.77% of the variance. This factor, called job dissatisfaction, clusters indicators in two dimensions, burnout factors and burnout syndrome. This factor compiles items that refer to the balance between job expectations and reality, and how much enjoyment the individual finds in the job [21]. This coincides with the proposal made by Moreno et al. [35], in their questionnaire for evaluating professional burnout in doctors, where a factor referring to the loss of job expectations was included. Similarly, the second factor, made up of four items, groups indicators corresponding to the relationship the worker establishes with fellow workers and superiors at work. This factor, which is called social climate, responds to a cluster that may be due to the importance in developing burnout of chronic stressful interpersonal situations in the workplace [6]. The third factor grouped seven items, which in the original questionnaire were in the burnout syndrome scale, except for one, which was on the consequences scale. The cluster of these items is called the personal impact factor and refers to the direct consequences exhaustion has on different areas of an employee’s life. 

Finally, the fourth factor, called motivational exhaustion, combines five items that in the original model were part of the burnout syndrome scale. The questions grouped under this factor of motivational exhaustion refer to the absence of job growth and stimulation for development in the job position. These are aspects that promote work demotivation, generate distress [17], and are among the challenges health care personnel face most frequently [18].

Although this four-factor model showed adequate fit, after making the corresponding respecifications according to theoretical and statistical criteria, items 2, 16, 3, 13, 17, and 11 were eliminated, so all 21 items in the original questionnaire were not retained. The items that were finally kept in each of the factors in the Brief Burnout Questionnaire Revised were items 6, 14, 19, and 20 for job dissatisfaction; 4, 8, and 9 in the social climate factor; 1, 7, 15, and 21 in personal impact; and 5, 10, 12, and 18 in the motivational exhaustion factor. 

The model fit improved considerably this way, and also showed consistency in the validation sample. Configural and metric invariance of the model across the type of job (permanent/temporary) is also assumed, and invariance in all cases (configural, metric, scalar, and strict) across gender. Given the divergence found when clustering items, inquiry into the adequacy of the structure reported by the CBB-R for nursing personnel will have to continue. The multifactorial construct of burnout shown here, which differs from the one reported by the authors of the original study, shows the need for further study of the internal structure of the evaluation instruments in this construct in the population studied [50]. In the process of adapting and validating instruments for certain populations, it must be known whether the factor structure coincides or not with the terms of the original version, as the job characteristics of each sample partly moderate the conditions where burnout appears. The proposed model also includes an analysis of all the burnout risk and protection factors now known as found in the theoretical review. 

## 5. Conclusions

The Brief Burnout Questionnaire Revised for health care personnel in nursing has favorable psychometric properties. The internal consistency of the total scale and each of the factors is adequate, therefore the general fit is acceptable. However, it is recommended that goodness and fit of the model continue to be analyzed to test the psychometric properties of the instrument in other groups, since this model of burnout can be applied to all care professionals.

This new evaluation model based on the CBB questionnaire adapted as an instrument for evaluation of the syndrome in health care personnel is intended to approach even closer to knowledge of burnout, exploring the different facets that comprise it. Thus, the purpose of validating the instrument was to approach burnout’s present reality. As a syndrome linked to the work environment of individuals, burnout will continue to evolve with it, accumulating new factors workers must cope with that may also lead to burnout.

## Figures and Tables

**Table 1:** Factor structure, communalities (h2), eigenvalues, Cronbach’s alpha, and percentage of explained variance (*n* = 1236). Extraction method: principal component analysis.

Principal component analysis	F1	F2	*h* ^2^
Item 2	0.56	0.63	0.53
Item 4	0.65		0.42
Item 6		0.79	0.63
Item 8	0.81		0.66
Item 9	0.79		0.62
Item 10		0.55	0.31
Item 14		0.68	0.48
Item 16	0.78		0.63
Item 20		0.80	0.64
Eigenvalue	3.56	1.37	
Percentage explained variance	39.51	15.22	54.73
Kaiser–Meyer–Olkin	0.85		
Barlett’s sphericity	*χ^2^*_(36)_ = 3019.35, *p* < 0.000		
Cronbach’s alpha	0.75	0.73	0.79

Note: Items are listed in decreasing order by saturation. Visualization coefficient > 0.40. F1: organization; F2: task.

**Table 2:** Factor structure, communalities (*h*^2^), eigenvalues, Cronbach’s alpha, and percentage of explained variance (*n* = 1236). Extraction method: principal component analysis.

Principal component analysis	F1	F2	F3	*h* ^2^
Item 1	0.76			0.68
Item 3			0.72	0.58
Item 5		0.49		0.27
Item 7	0.60			0.48
Item 11			0.44	0.37
Item 12		0.50		0.33
Item 15	0.72			0.66
Item 18		0.28		0.11
Item 19		0.30		0.17
Eigenvalue	3.38	0.98	0.96	
Percentage explained variance	31.93	4.99	3.74	40.66
Kaiser–Meyer–Olkin		0.84		
Barlett’s sphericity		*χ*^2^_(36)_ = 2569.33, *p* < 0.000		
Cronbach’s alpha	0.81	0.49	0.57	0.76

Note: Items are listed order by saturation in decreasing. Visualization coefficient >0.40. F1: emotional exhaustion; F2: lack of accomplishment; F3: depersonalization.

**Table 3:** Factor structure, communalities (*h*^2^), eigenvalues, Cronbach’s alpha, and percentage of explained variance (*n* = 1236). Extraction method: principal component analysis.

Principal component analysis	F1	F2	F3	F4	*h* ^2^
Item 1	0.56	0.41	0.65		0.61
Item 2	0.59	0.58			0.55
Item 3			0.62	0.45	0.49
Item 4		0.65			0.43
Item 5	0.43			0.57	0.41
Item 6	0.70			0.56	0.64
Item 7	0.44		0.62		0.51
Item 8		0.80			0.66
Item 9		0.78			0.62
Item 10				0.61	0.38
Item 11			0.40	0.67	0.51
Item 12	0.44			0.62	0.47
Item 13			0.78		0.62
Item 14	0.56		0.41		0.40
Item 15	0.56		0.70		0.66
Item 16		0.77	0.41		0.64
Item 17			0.66		0.47
Item 18				0.53	0.30
Item 19	0.66				0.44
Item 20	0.76				0.64
Item 21			0.62		0.46
Eigenvalue	6.67	1.76	1.39	1.06	
Percentage explained variance	31.77	8.41	6.64	5.05	51.86
Kaiser–Meyer–Olkin	0.92				
Barlett’s sphericity			*χ*^2^_(210)_ = 8449.54, *p* < 0.000		
Cronbach’s alpha	0.74	0.75	0.82	0.59	0.88

Note: Items are listed in decreasing order by saturation. Visualization coefficient >0.40. F1: job dissatisfaction; F2: social climate; F3: personal impact; F4: motivational exhaustion.

**Table 4:** Fit indices for the models proposed (calibration sample; *n* = 605).

Model	*χ*^2^ (*df*)	*χ*^2^/*df*	CFI	TLI	RMR	Est.	RMSEA	
							CI 90%	
							Bel.	Abv.
Original CBB model	931.446 (179)	5.204	0.822	0.791	0.042	0.083	0.078	0.089
Unidimensional CBB model	1305.043 (189)	6.904	0.735	0.706	0.059	0.099	0.094	0.104
Proposed CBB model	664.676 (183)	3.632	0.886	0.869	0.044	0.066	0.061	0.071
Proposed CBB-R model	176.497 (84)	2.101	0.965	0.956	0.027	0.043	0.034	0.052

Note: CFI, comparative fit index; TLI, Tucker–Lewis index; RMSEA, root mean square error of approximation; CI, confidence interval; *df*, degrees of freedom; Est., estimation; Bel., below; Abv., above. CBB: CBB-R (Revised CBB Model).

**Table 5:** Multigroup analysis of variance by type of contract (permanent/temporary) and gender (male/female).

Model	*χ^2^*	*gl*	*χ^2^/gl*	*Δχ^2^*	CFI	ΔCFI	IFI	RMSEA (CI 90%)
M0a (permanent)	376.265 (*p* = 0.000)	168	2.239		0.960		0.961	0.032 (0.027–0.036)
M0b (temporary)	417.761 (*p* = 0.000)	179	2.333		0.955		0.955	0.033 (0.029–0.037)
M1 (base model set)	505.309 (*p* = 0.000)	194	2.604		0.941		0.941	0.036 (0.032–0.040)
M2 (FS)	544.696 (*p* = 0.000)	209	2.606	39.387	0.936	0.005	0.936	0.036 (0.032–0.040)
M3 (FS + Int)	376.265 (*p* = 0.000)	168	2.239	129.044	0.960	0.024	0.961	0.032 (0.027–0.036)
M4 (FS + Int + Err)	376.265 (*p* = 0.000)	168	2.239	129.044	0.960	0.024	0.961	0.032 (0.027–0.036)
M0a (male)	383.819 (*p* = 0.000)	168	2.284		0.959		0.960	0.032 (0.028–0.037)
M0b (female)	407.567 (*p* = 0.000)	179	2.276		0.957		0.957	0.032 (0.028–0.036)
M1 (base model set)	446.771 (*p* = 0.000)	194	2.302		0.952		0.953	0.032 (0.029–0.036)
M2 (FS)	474.727 (*p* = 0.000)	209	2.271	27.956	0.950	0.002	0.950	0.032 (0.028–0.036)
M3 (FS + Int)	383.819 (*p* = 0.000)	168	2.284	62.952	0.959	0.009	0.960	0.032 (0.028–0.037)
M4 (FS + Int + Err)	376.265 (*p* = 0.000)	168	2.284	62.952	0.959	0.009	0.960	0.032 (0.028–0.037)

Nota FS = factor saturation; Int = intercepts; Err = error.
